# Identification of Potential Drug Targets for Antiplatelet Therapy Specifically Targeting Platelets of Old Individuals through Proteomic Analysis

**DOI:** 10.3390/biomedicines11112944

**Published:** 2023-10-31

**Authors:** Seung Hee Lee, Suyeon Cho, Jong Youl Lee, Jung Yeon Hong, Suji Kim, Myong-Ho Jeong, Won-Ho Kim

**Affiliations:** Division of Cardiovascular Disease Research, Department of Chronic Disease Convergence Research, Korea National Institute of Health, Cheongju-si 28159, Republic of Korea; sy59291@korea.kr (S.C.);

**Keywords:** platelets, aging, antiplatelet therapy, proteomics

## Abstract

Aging is a growing problem worldwide, and the prevalence and mortality of arterial and venous thromboembolism (VTE) are higher in the elderly than in the young population. To address this issue, various anticoagulants have been used. However, no evidence can confirm that antithrombotic agents are suitable for the elderly. Therefore, this study aims to investigate the platelet proteome of aged mice and identify antithrombotic drug targets specific to the elderly. Based on the proteome analysis of platelets from aged mice, 308 increased or decreased proteins were identified. Among these proteins, three targets were selected as potential antithrombotic drug targets. These targets are membrane proteins or related to platelet function and include beta-2-glycoprotein 1 (β2GP1, ApolipoproteinH (ApoH)), alpha-1-acid glycoprotein2 (AGP2, Orosomucoid-2 (Orm2)), and Ras-related protein (Rab11a).

## 1. Introduction

Aging is considered a significant risk factor for CVD and thrombosis. According to the 2022 World Health Organization report, the global population of individuals aged 60 and older is projected to reach 2.1 billion by 2050. The number of people aged 80 years or older is expected to triple between 2020 and 2050, which could reach 426 million. In Korea, due to the rapid aging of the population, the proportion of elderly individuals aged over 65 years is expected to reach 17.5% of the total population in 2022, 30.1% in 2035, and 40.1% in 2050 (Statistics Korea 2022). As the population ages, a significant rise in the incidence of major chronic diseases, such as heart disease, cerebrovascular disease, diabetes, and thrombosis in the blood vessels has been observed. This has led to an increased burden on medical care. Additionally, the risk of platelet aggregation is increased in cardiovascular and cerebrovascular diseases, diabetes, and other conditions, which can lead to mortality in older adults.

Platelets are small, anucleate cells in the blood that regulate hemostasis and thrombosis. Platelets are highly associated with thrombotic disease, and their levels are known to decrease with age [1]. The Third National Health and Nutrition Examination Survey (12,142 participants) reported that the number of platelets in 1 μL of blood was 10 × 10^3^ in individuals aged 60–69 years, which is very low compared with 20 × 10^3^ in those aged 20–59 years. In another study, a comparison was made among 33,258 participants, which indicated that platelet number decreases with age [1]. Bleeding time was also found to reduce in older adults. Meade et al. reported an increase in platelet cohesion by 8% for every 10 years of age. Moreover, the elderly are expected to have an increased secretion of b-thrombomodulin, platelet factor 4 (PF4), and other platelet aggregation factors, contributing to increased cohesion. VTE, including deep vein thrombosis and pulmonary embolism, is expected in the elderly [2]. This condition is rare in the young population and is reported to increase by 1% annually in the elderly population [3]. This is believed to be due to the increased platelet aggregation in elderly individuals and the increase in platelet aggregation factors in the blood. 

The COVID-19 pandemic has increased the prevalence of venous thromboembolism, arterial thrombosis, and thrombotic microangiopathy, showing a high mortality rate worldwide. These disease forms are often related to underlying conditions, such as diabetes and obesity, which are major risk factors for CVD and are commonly observed in the elderly population. Therefore, platelet-related disorders in diabetic and obese elderly individuals are considered significant contributing factors to the morbidity and mortality of COVID-19 in this population [4].

This study aimed to identify new potential antithrombotic agents by examining proteins that exhibit increased expressions specifically in super-aged mice platelets.

## 2. Material and Methods

### 2.1. Preparation of Aged Mice

All mice had a C57Bl/6 background. The animals were housed at Korea NIH (LML-KCDC-11-2-26). All experiments were performed in accordance with the guidelines and regulations as outlined by the Korea Disease Control and Prevention Agency (KDCA) Animal Care and Use Committee (IACUC) under the approved protocol by KDCA-IACUC-21-028. For each animal study, consecutive bred mice were included. No significant variance was observed within the groups since the mice were from the same genetic background and were often siblings. Any differences would therefore be directly related to treatment or modification.

### 2.2. CD62p ELISA

Serum CD62p was calculated by Mouse Premixed Multi analyst Kit (LXSAMSM-07) according to the manufacturer’s instructions (R&D Biosystems, MN, USA). 

### 2.3. Platelet Isolation 

Blood (0.7–1 mL) was directly aspirated from the right cardiac ventricle into 1.8% sodium citrate (pH 7.4) in young and old mice. Citrated blood from several mice with identical genotypes was pooled and diluted with equal HEPES/Tyrode buffer volume. PRP was prepared by centrifugation at 100 g for 10 min and then used for proteomic analysis, immunostaining, and Western blotting. All mice had a C57Bl/6 background.

### 2.4. Western Blotting

We used the standard Western blot analysis protocols [5] and specific individual antibodies and dilutions (fibronectin: Abcam ab2413 (1:1000), CD42b: Abcam ab183345 (1:1000), ApoH: Proteintech 66074 (1:1000), and Orm2: Abbexa abx101108 (1:1000).

### 2.5. Immunostaining

Washed platelets (1 × 10^6^ cells/mL) were allowed to settle on glass-bottomed dishes for 1 h before fixing with 4% paraformaldehyde solution (Biosesang, Gyeonggi-do, Korea). The platelets were washed for 2 × 5 min in PBS and permeabilized for 5 min in 0.25% Triton X-100/PBS. They were blocked for 60 min in 10% bovine serum albumin (BSA)/PBS at 37 °C, incubated in 3% BSA/PBS/primary antibody for 2 h at 37 °C. Subsequently, they were washed for 5 × 2 min in PBS, followed by an additional incubation for 45 min at 37 °C in secondary antibody/3% BSA/PBS. Moreover, antibodies for ORM2 Abbexa abx101108 (1:1000) and Rab11a Invitrogen 71-5300 (1:1000), and tubulin (1:000) were used. The stained platelets were observed using an Olympus FV-3000 microscope with a 100× oil immersion lens.

### 2.6. Proteomics

1. Protein digestion: the samples were suspended in lysis buffer (8 M urea–0.1 M Tris-HCl buffer, pH 8.5) and protease inhibitor cocktail, followed by sonication for 20 min at 15 °C using Covaris S2 Focused Ultrasonicator (Covaris, Woburn, MA, USA). The Pierce BCA Protein Assay Kit (Thermo Fisher Scientific, IL, USA) quantified the protein concentration. The digestion step was performed using filter-aided sample preparation on a Microcon 30 K centrifugal filter device (Millipore, Billerica, MA, USA). Each sample was reduced by incubating with Tris (2-carboxyethyl) phosphine (TCEP) at 37 °C for 30 min and alkylated with iodoacetic acid (IAA) at 25 °C for an hour in dark conditions. After washing with lysis buffer and 50 mM ammonium bicarbonate (ABC) sequentially, the proteins were digested with trypsin (enzyme to protein ratio of 1:50; *w*/*w*) at 37 °C for 18 h. The resulting peptide mixtures were transferred into new tubes, and trypsin was inactivated by acidifying with 15 μL of formic acid (Honeywell, Charlotte, NC, USA). The digested peptides were desalted using C18 spin columns (Harvard Apparatus, Holliston, MA, USA), and the peptides were eluted with 80% acetonitrile in 0.1% formic acid in water.
2. LC–MS/MS analysis: The prepared samples were resuspended in 0.1% formic acid in water and analyzed using a Q-Exactive Orbitrap hybrid mass spectrometer (Thermo Fisher Scientific, Waltham, MA, USA) along with an Ultimate 3000 system (Thermo Fisher Scientific, Waltham, MA, USA). We used a 2 cm × 75 μm ID trap column packed with 3 μm C18 resin and a 50 cm × 75 μm ID analytical column packed with 2 μm C18 resin for the peptides depending on the peptides’ hydrophobicity. The mobile phase solvents consisted of (A) 0.1% formic acid in water and (B) 0.1% formic acid in 90% acetonitrile, whereas the flow rate was fixed at 300 nL/min. The gradient of the mobile phase was as follows: 4% solvent B for 14 min, 4%–15% solvent B for 61 min, 15%–28% solvent B for 50 min, 28%–40% solvent B for 20 min, 40%–96% solvent B for 2 min, holding at 96% of solvent B for 13 min, 96%–4% solvent B for 1 min, and 4% solvent B for 24 min. A data-dependent acquisition method was adopted, and the top 10 precursor peaks were selected and isolated for fragmentation. Ions were scanned in high resolution (70,000 in MS1, 17,500 in MS2 at m/z 400), and the MS scan range was 400–2000 m/z in both the MS1 and MS2 levels. Precursor ions were fragmented with a normalized collisional energy of 27%. Dynamic exclusion was set to 30 s.
3. Proteome data analysis: Thermo MS/MS raw files of each analysis were searched using the Proteome Discoverer™ software (ver. 2.5), and the mouse database was downloaded from UniProt. The appropriate consensus workflow included a peptide-spectrum match validation step and a SEQUEST HT process for detection as a database search algorithm. The search parameters were set up as follows: 10 ppm of tolerances of precursor ion masses, 0.02 Da fragment ion mass, and a maximum of two missed cleavages with trypsin enzyme. The dynamic modification on the peptide sequence was as follows: static carbamidomethylation of cysteine (+57.012 Da), variable modifications of methionine oxidation (+15.995 Da), acetylation of protein N-term (+42.011 Da), and carbamylation of protein in N-term (+43.0006 Da). After searching, the data results below 1% of FDR were selected and filtered for at least six more peptide lengths. Precursor abundance calculation was based on intensity. Fold change was calculated in the protein abundance-based ratio. Furthermore, the *p*-values were calculated for the reported quantification ratios using a *t*-test based on the background.

### 2.7. Statistics

Each experiment was carefully designed and analyzed with standard and accepted statistical analyses. Where appropriate, all data are expressed as mean ± SD or ± SE. An ordinary one-way ANOVA was performed to compare two groups as outlined with the separate experiments. A *p*-value of <0.05 was considered statistically significant. Analysis was performed using Prism software version 10 (GraphPad Software, Inc., La Jolla, CA, USA).

## 3. Results

### 3.1. Identification of Proteins with Increased or Decreased Expression in Aged Mice Platelets

**Figure 1:**
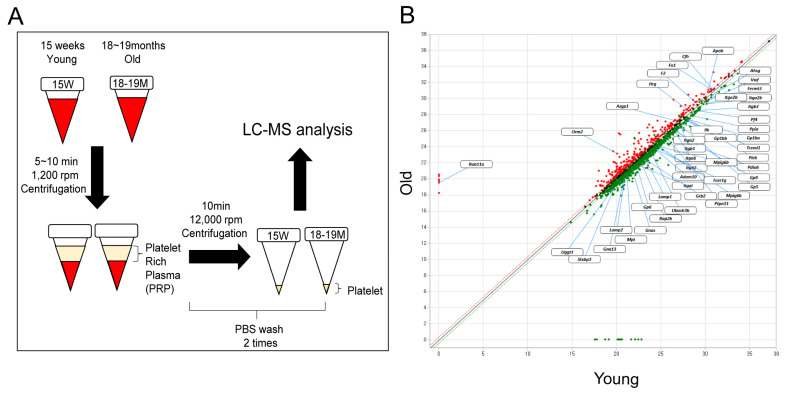
Proteomic analysis of young and aged mice platelets. (**A**) Experimental flow chart from blood drawn for LC-Mass analysis. (**B**) Comparison of upregulated and downregulated protein platelets from old and young mice.

**Figure 2:**
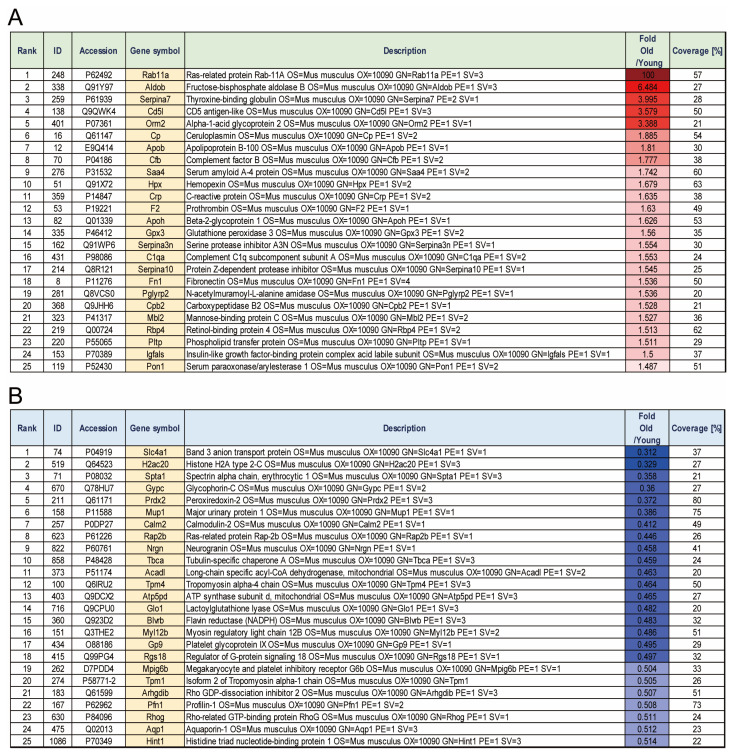
Top 25 upregulated or downregulated proteins in platelets from old mice. (**A**) Top 25 upregulated proteins in platelets of aged mice. (**B**) Top 25 downregulated proteins in platelets of young mice.

Platelets were isolated from 15 weeks (human age: 20–30) and 18–19 months (human age: older than 70) mouse blood and the expression changes of the whole protein were analyzed by LC-Mass (Figure 1). Each group consists of three individuals and was separately analyzed by LC-Mass. Compared with platelets in young mice, the expression changed 1.3 times in aged mice platelets, and the analysis was based on proteins with a peptide coverage of 20% or more. 308 proteins were increased (52 proteins) or decreased (256 proteins) 1.3-fold in aged mouse platelets compared with the 15-week-old mouse platelets. Among them, the top 25 are represented in Figure 2.

**Figure 3:**
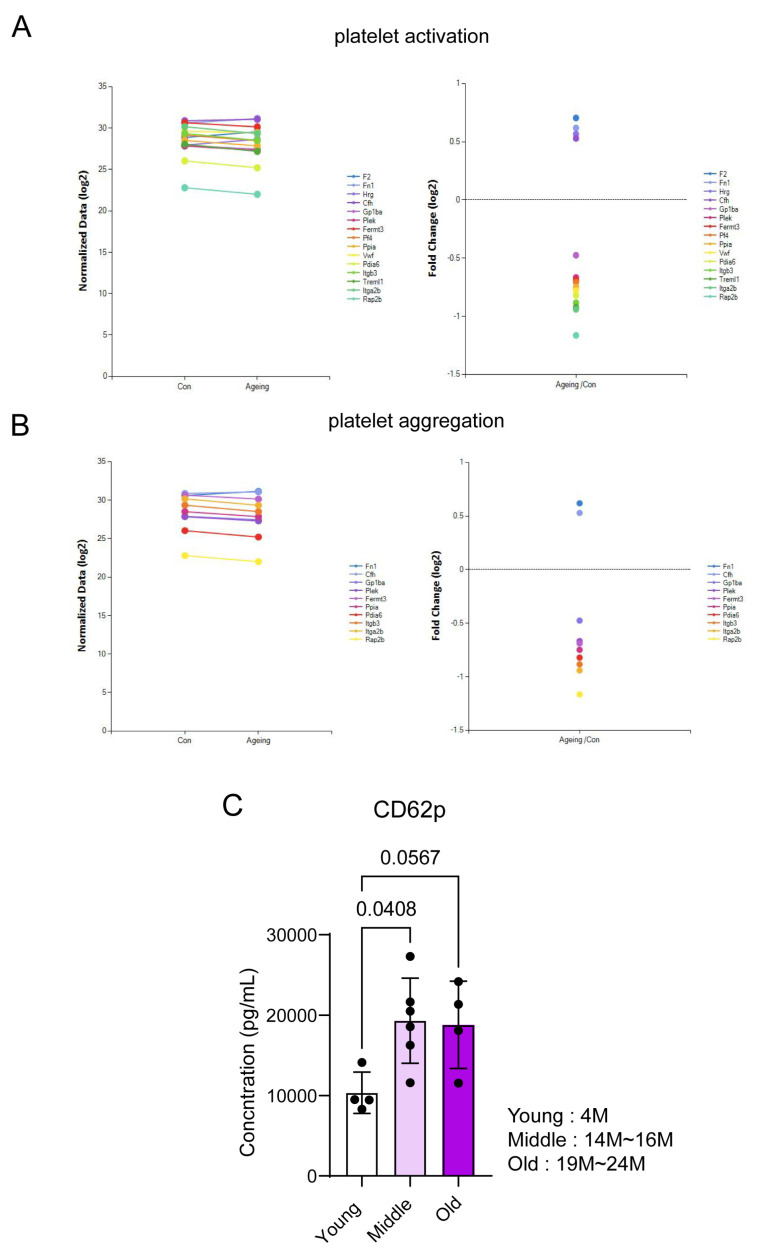
Secretion of proteins related to platelet activity and aggregation in aged mice. (**A**) Platelet-activation-related proteins are downregulated in platelets from aged mice. (**B**) Platelet-aggregation-related proteins are downregulated in platelets from aged mice. (**C**) Increased CD62p (p-Selectin) levels in plasma from 14-month-old mice.

Regarding the total 308 protein changes, the tendency of platelet activity and expression of various proteins associated with aggregation in aged mice platelets decreased inside platelets; maybe these were already secreted in plasma (Figure 3A,B). Furthermore, the platelet activation marker CD62p increased more in 14- and 24-month-old mice than in young mice plasma (Figure 3C). These results confirmed that the plasma from aged mice contains many proteins related to platelet activity and aggregation, unlike the proteome of aged mice platelets.

**Figure 4:**
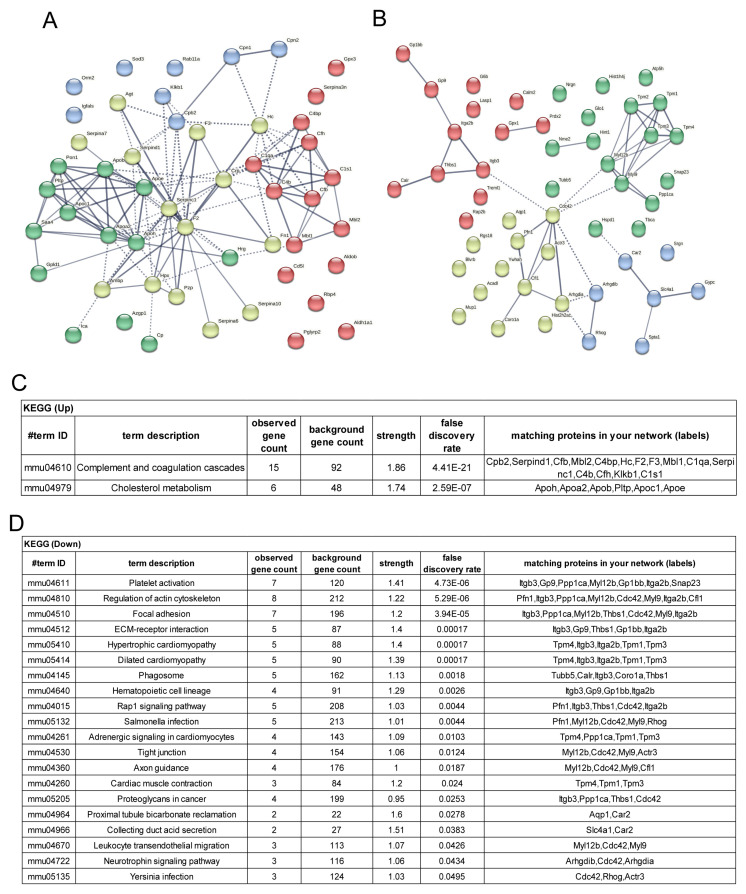
The STRING and KEGG analysis among up or downregulated proteins. (**A**,**C**). STRING and KEGG analysis in upregulated proteins. (**B**,**D**). STRING and KEGG analysis in downregulated proteins.

To determine the function of proteins that showed significant increases or decreases in aged mice platelets, string analysis was conducted using 50 proteins that showed the most significant changes in expression levels (Figure 4). According to the KEGG pathway analysis of the top 50 proteins that were highly increased, many of them were related to complement and coagulation cascades and cholesterol metabolism. As for the gene ontology (GO) analysis, the highly increased proteins were associated with lipoprotein function and extracellular space. Many of the top 50 highly decreased proteins were associated with platelet activation, focal adhesion, ECM receptor interaction, and Leukocyte trans endothelial migration-related proteins (Figure 4).

**Figure 5:**
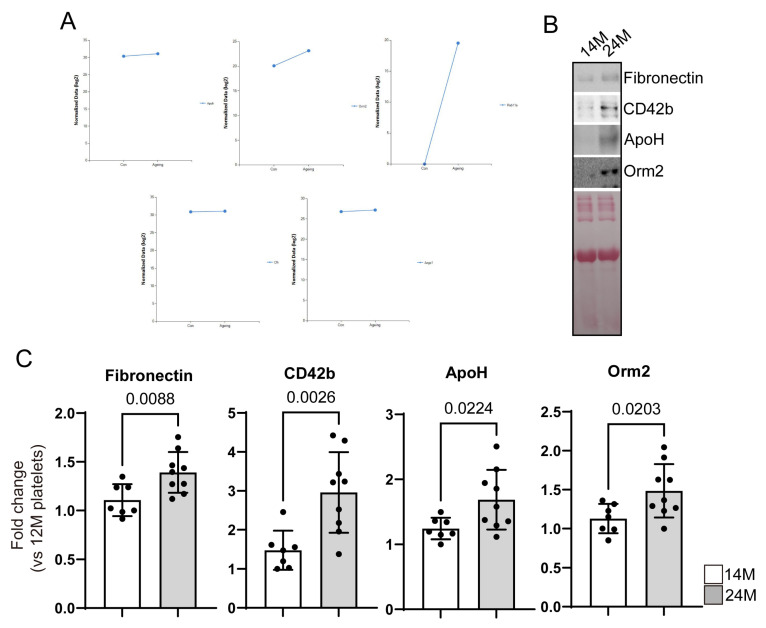
Expression of selected targets in young and aged mice platelets. (**A**) Expression of ApoH, Orm2, Rab1, cfh, and Azgp1/ZAG from proteomic analysis. (**B**) Western blot analysis of platelet activation markers (Fibronectin and CD42b), and selected targets of 14-month-old mice platelets (#1–7) and 24-month-old mice platelets (#1–9). Ponceau’s staining served as loading control. (**C**) Quantification of fibronectin, CD42b, ApoH, and Orm2 signal intensity. The *p*-values are analyzed by one-way ANOVA.

Based on the results of the proteome analysis, 25 significantly upregulated proteins were identified. Among them, proteins related to platelet activation, aggregation, integrin, glycoprotein, and other platelet functions were determined as potential targets of antithrombotic agents. The selected targets were only expressed in aged mice platelets. The following proteins were selected as potential targets: β-2-glycoprotein1 (β2GP1, ApolipoproteinH (ApoH)), complement factor H (Cfh), Zinc-α-2-glycoprotein (Azgp1/ZAG), alpha-1-acid glycoprotein 2 (AGP2, Orosomucoid-2(Orm2)), and Ras-related protein (Rab11a) (Figure 5A).

### 3.2. Reconfirmation of Target Protein Expression in Aged Mice Platelets

**Figure 6:**
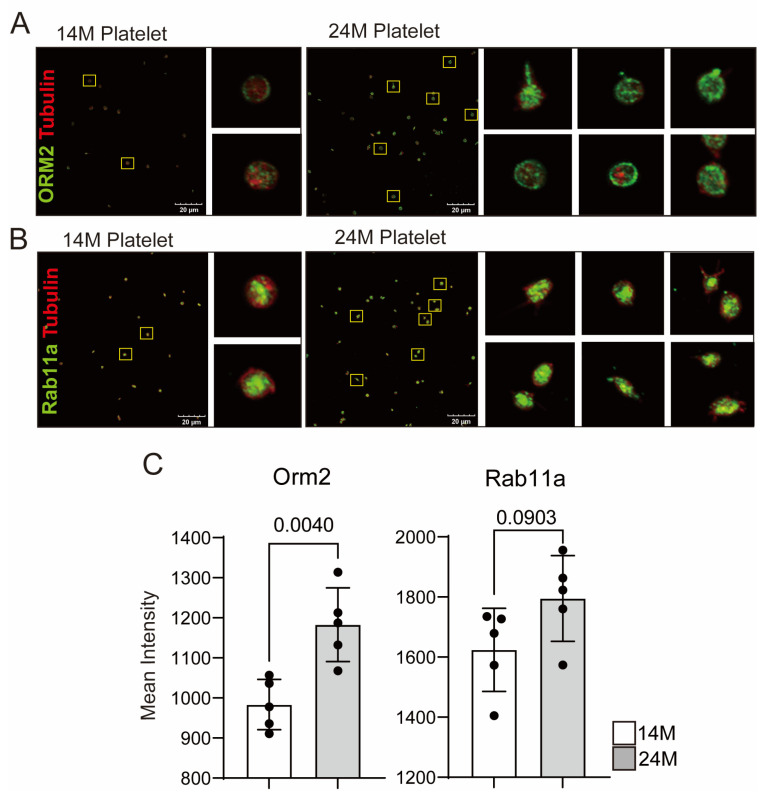
The expression level of Orm2 increased in aged mice platelets. (**A**,**B**). Confocal microscopy images using Orm2 and Rab11a specific Abs in 14- and 24-month-old mice platelets. (**C**). Quantification of Orm2 and Rab11a relative signal intensity. The *p*-values are analyzed using a one-way ANOVA.

We used platelets from 15-week-old/18- to 19-month-old mice to verify which proteins were related to platelet functions in aged mice. As we mentioned, five targets were selected and platelets were used from 14-month-old (human age: 40–50) and 24-month-old (human age: late 70s, super-aged) mice for WB and immunostaining to confirm whether the identified proteins show specific changes in aged mice, especially in super-aged mice. We confirmed the expression changes in target proteins in the two groups, and immunostaining was performed to investigate the direct expression in platelets and the expression on the platelet membrane (Figure 5 and Figure 6). Among the proteins that have increased in aged mice platelets based on the proteome analysis, only the expressions of Fn1, ApoH, and Orm2 significantly increased (Figure 5B,C). The other targets showed a tendency to increase in aged mice platelets, which was consistent with the increased expression of the platelet activation marker CD42b (platelet glycoprotein GPIb, receptor of von Willebrand factor), but no statistical significance was observed owing to large individual differences.

According to the results of the immunostaining performed on platelets from 14- and 24-month-old mice, Orm2 was the only protein that showed a significant increase in expression in aged mice platelets, with an increased expression observed around the platelet membrane (Figure 6). Although CFH, ZAG, and Rab11a showed no significant differences in the immunostaining results, they showed a moving pattern toward the platelet membrane. 

### 3.3. Potential Targets of Antithrombotic Agent

We attempted to investigate the correlation between these targets (ApoH, Orm2, and Rab11a) and an increased activity of platelets. Through the literature research on the obtained targets, we found that many proteins are related to the function of growing microparticle secretion in platelets. Furthermore, the possibility of using proteins that increase in super-aged mice platelets as targets for antithrombotic agents was investigated through various literature searches.

1. Beta-2-glycoprotein1 (β2GP1, Apolipoprotein H (ApoH)

Apolipoprotein H, also known as beta-2-glycoprotein 1, is a multifunctional glycoprotein. ApoH is a 43–50 kDa single-chain glycoprotein highly expressed in the liver and endothelial cells, lymphocytes, astrocytes, and neurons [6,7,8] and is associated with lipid metabolism, coagulation, apoptosis, host defense, inflammation, and atherogenesis [9,10,11,12]. ApoH is initially produced in hepatocytes and is detected in various human tissues, particularly in the subendothelial and intima-media regions of arteriosclerosis [13,14], and it is known to perform a significant function in regulating thrombosis and pregnancy morbidity [14]. A comparison of plasma protein levels in middle-aged and older adults showed that ApoH is associated with adverse health outcomes [7]. In a platelet proteomic analysis of acute ischemic stroke patients, ApoH was expressed in platelets and associated with early-stage stroke [15]. It has been reported that ApoH is involved in platelet damage and hemostasis inhibition, which are significant factors in controlling the severity of COVID-19 infection [16].

Immunostaining for ApoH in aged mice platelets was not performed in this study. However, in WB analysis, a significant increase in expression was observed in platelets from aged mice compared with those from young mice (Figure 5). Although the function of ApoH is known in various cardiovascular diseases, its role still needs to be investigated further. Considering the limitations of this study, further research is required to investigate the increased expression of ApoH in super-aged mice platelets.

2. Alpha-1-acid glycoprotein2 (AGP-1, Orm2)

AGP-1/Orm2 is a positive acute phase glycoprotein that increases during an inflammatory response [17,18]. It is known to undergo 12–20 different glycosylations, and the glycosylation pattern can differ depending on whether the inflammation is acute or chronic [19,20,21,22,23,24,25]. Different glycoforms of AGP-1/Orm2 have also been shown to have other functions in platelets and neutrophils. For example, secreted AGP-1 in blood from the liver can inhibit platelet aggregation induced by PAF and ADP, while neutrophil-secreted nAGP-1 does not exhibit this function [26]. AGP-1/Orm2 has an antiheparin effect in the blood, which reduces its anticoagulation impact [27]. Increased levels of AGP-1/Orm2 have also been correlated with the increased incidence of ischemic stroke and carotid plaque [28]. AGP-1/Orm2 is also known to stabilize the function of the plasminogen activator inhibitor type 1, regulate platelet shape and activity, and ultimately induce thrombosis [29,30]. On the other hand, other reports have indicated that AGP-1/Orm2 induces platelet aggregation and exhibits protective effects in antithrombotic activity and ischemia-reperfusion injury by suppressing cell death and inflammation [31,32,33,34].

In COVID-19 pneumonia patients, neutrophil, platelet, and complement activation are induced, which are known to regulate the severity of the disease [35]. Acute inflammation response-related proteins, including ORM1, ORM2, SAA1, S100AB, S100A9, and Serpina3, are expressed in the blood of COVID-19 patients with high mortality after infection [35]. Another proteomic analysis reported that Orm2 expression is significantly decreased in gray platelet syndrome (GPS) compared with healthy platelets. However, the functional significance of this finding needs to be confirmed [36]. GPS is an autosomal recessive bleeding disorder caused by a deficiency of a-granules in platelets, which may also explain the decreased expression of Orm2 [36]. Another study reported that Orm1 and Orm2 in yeast are ER membrane proteins that regulate lipid homeostasis and protein quality control [37]. However, no studies have investigated the expression of AGP-1/Orm2 in platelets or its function in aged mice platelets. Further research is needed to investigate the potential use of AGP-1/Orm2 as a targeted antiplatelet therapy for the elderly population.

3. Ras-related protein Rab-11A (Rab11a)

Rab11a is a member of the Rab family of small GTPase proteins involved in membrane/protein trafficking. Among the Rabs, Rab11 is involved in various receptors, signal transduction, nerve cell function activation, and recycling of multiple proteins [38]. Reduction in expression due to LPS treatment leads to decreased vascular endothelial barrier function, which increases the risk of inflammatory diseases [39]. In pediatric patients with asthma, Rab11a expression tends to increase, promoting PDGF-BB dependent airway smooth muscle cell proliferation and migration [40]. However, its function in platelets requires further investigation. Rab11a is a protein that is expressed only in aged mice platelets. However, the results in WB and immunofluorescence staining suggested that Rab11a is also expressed in platelets from 14-month-old (human age: 40–50) mice, suggesting that Rab11a does not increase in young (15 weeks) mouse platelets but increases in middle age followed by old age. Further research is needed to confirm the Rab11a expression in platelets at different ages. If Rab11a expression increases with age, it may be considered a significant factor in platelet aging and it may be possible to inhibit platelet aging by utilizing this information.

## 4. Discussion

The main findings of our study are as follows: (1) platelet activation and aggregation proteins are secreted from aged mice platelets into the plasma, and (2) ApoH, Orm2, and Rab11a have been identified as potential therapeutic targets.

In aged mice platelets, many proteins decrease overall compared with young platelets, and many of these proteins are associated with platelet activation. Although the expression of many proteins associated with platelet activation and aggregation decreased (Figure 3), the literature indicated that vascular thrombosis increases as aging progresses [41]. This phenomenon can be explained by the increased platelet activation and aggregation proteins secreted from aged mice platelets into the plasma, which can increase the activation of the surrounding platelets. When the platelets are activated, more than 300 proteins are secreted, with CD62p being one of the secreted proteins. Moreover, elevated levels of CD62p in the blood can predict platelet activity [42]. Previously, the level of plasma CD62p was reported to increase in cases of malaria infection and stroke [43]. Our results confirmed that the amount of CD62p in mice plasma rose from 14 months to the super aged condition. Additionally, based on these results, an increase in platelet activity in aged mice can be predicted.

There is a higher decrease in protein expression levels in aged mice platelets due to increased secretion. The proteomics results confirmed that the amounts of Pf4 and vWf decreased in aged mice platelets. Despite an observed reduction in the expression of proteins related to platelet activity in aged mice, it can be assumed that proteins controlling platelet aggregation are secreted externally to potentially enhance the aggregation of other platelets. This phenomenon could support the idea of increased platelet hyperaggregability during the aging process. However, the hyperactivity concept in old platelets needs to be investigated further, and the likelihood of involvement of targets, other than known factors, must be determined. We aimed to identify the proteins that are increased explicitly in aged mice platelets by analyzing young and old mouse platelet proteins.

The proteins ApoH, Orm2, and Rab11a have been identified as potential therapeutic targets. Although the precise mechanisms by which these proteins function in aged platelets were previously unknown, ApoH is recognized for its established role in activating the coagulation cascade by generating antiphospholipid antibodies. Therefore, further investigation highlights the potential of ApoH as an inhibitory target for regulating aged mouse platelet function. Furthermore, the dramatic increase in expression of Rab11a during the initial stages of aging not only holds potential as an antiplatelet therapeutic target but also plays a significant role in governing platelet function in aging mice. The observed elevation in expression of Orm2 and enhanced membrane translocation in aged mouse platelets (Figure 4 and Figure 5) suggest that Orm2 is a potential antiplatelet therapeutic target. Several reports have indicated substantial induction or reduction in Orm2 expression in diverse diseases. However, reports of Orm2 in platelets are infrequent. Therefore, it is essential to conduct comprehensive research about the roles of Orm2 in platelets across various conditions. These endeavors can identify Orm2 as a promising target for platelet-related disorders. Consequently, it is anticipated to substantially contribute to developing effective treatments for diverse platelet-related conditions in the future.

This study found that although the reactive aggregation of platelets themselves is reduced in the elderly, there are several platelet aggregation factors in the blood. Considering these characteristics, it was confirmed that an antithrombotic agent should be used in older people. In addition, Orm2, whose expression is particularly increased in the platelets of elderly people and moves to the platelet membrane, was suggested as a target for an anti-hemostatic agent tailored to the elderly.
